# Gastric Inflammatory Fibroid Polyp: A Rare Incidental Finding on Endoscopy

**DOI:** 10.1093/jcag/gwaa030

**Published:** 2021-06-04

**Authors:** Robert Bechara, David Hurlbut, Andrea Grin

**Affiliations:** 1 Department of Medicine, Division of Gastroenterology, Queens University, Kingston Health Sciences Center, Kingston, Ontario, Canada; 2 Department of Pathology and Molecular Medicine, Queens University, Kingston Health Sciences Center, Kingston, Ontario, Canada

**Keywords:** Endoscopic submucosal dissection, ESD, Gastric polyp, Inflammatory fibroid polyp, Submucosal tumor

**Figure 1.**
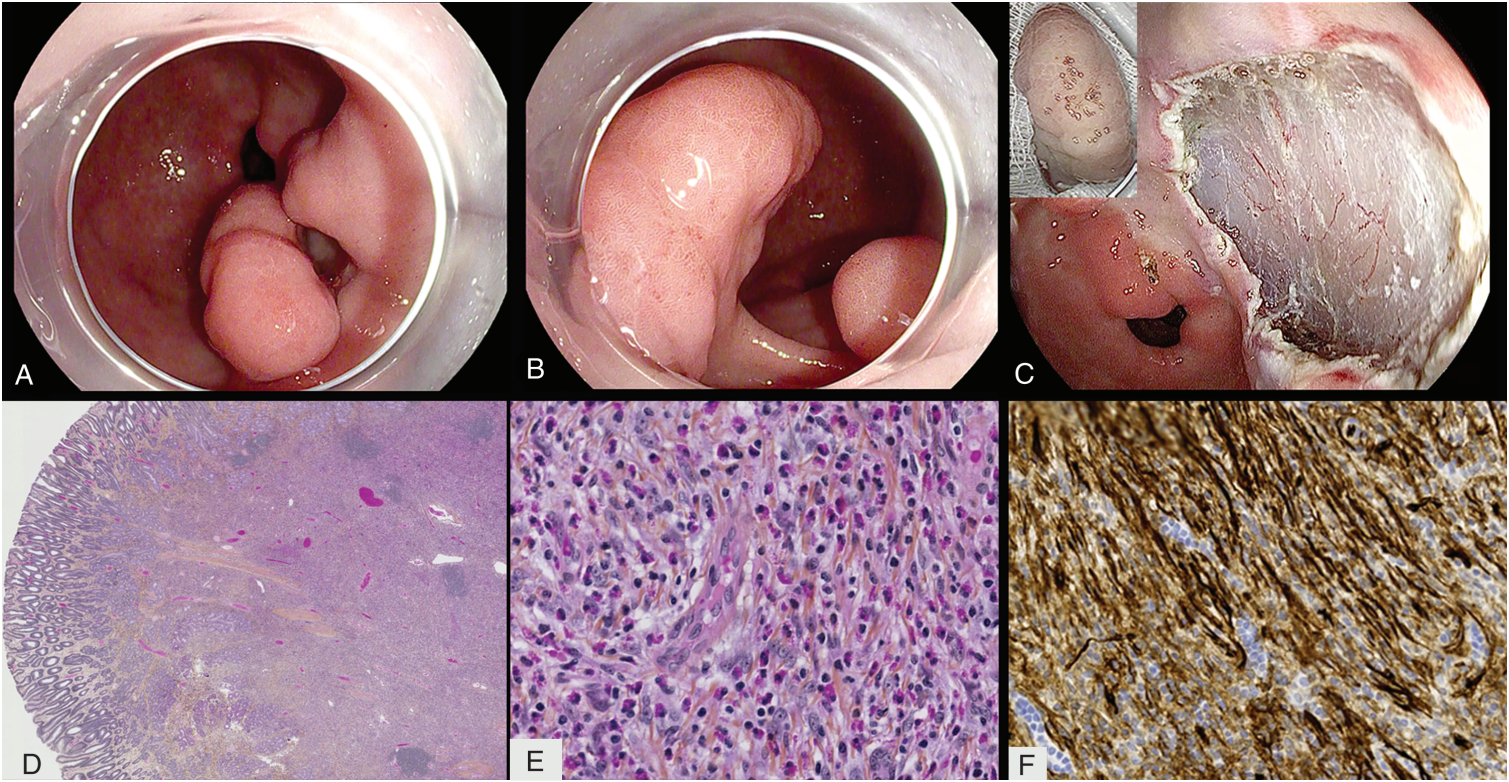
Endoscopic and histologic views of inflammatory fibroid polyp. (**A**) Distant view demonstrating a Paris 1sp lesion in the antrum. (**B**) Closer view showing normal overlying antral mucosa. (**C**) Final endoscopic submucosal dissection defect with the gross specimen (top left insert). (**D**) At low power, there is a cellular proliferation filling the submucosa and extending into the basal portion of the mucosa. (**E**) On high power, cytologically bland spindle cells mixed with small blood vessels and eosinophil-rich mixed inflammation are seen. (**F**) The stromal cells are positive for CD34 by immunohistochemistry. CD117 and DOG-1 are negative.

A 73-year-old man presented for upper endoscopy due to right upper quadrant pain that was subsequently diagnosed as myofascial pain. Incidentally, endoscopy demonstrated a 3-cm antral submucosal tumour with a sub-centimeter satellite nodule (Figure 1A and B). The patient subsequently had endoscopic ultrasound + fine needle aspiration that demonstrated a hypoechoic lesion arising from the muscularis mucosa. Fine needle aspiration was non-diagnostic. Considering that the lesion met size criteria, a plan for resection was made. Given the unknown pathology, the lesion was excised via endoscopic submucosal dissection to ensure an R0 resection. Final pathology demonstrated an inflammatory fibroid polyp (Figure 1D–F). Inflammatory fibroid polyps are rare gastric lesions that makeup ~0.1% of encountered gastric polyps (1). These are usually incidental findings on upper endoscopy. However, they can result in bleeding, gastric outlet obstruction, epigastric pain or recurrent nausea, and vomiting (2,3). Although rare, IFPs should be included in the differential for gastric submucosal tumours.
